# Study and characterization of morphogeometric parameters to assist diagnosis of keratoconus

**DOI:** 10.1186/s12938-018-0564-7

**Published:** 2018-11-20

**Authors:** Francisco Cavas-Martínez, Daniel G. Fernández-Pacheco, Dolores Parras, Francisco J. F. Cañavate, Laurent Bataille, Jorge Alió

**Affiliations:** 1 Department of Graphical Expression, Technical University of Cartagena, C/Doctor Fleming s/n, 30202 Cartagena, Murcia, Spain; 2 Research and Development Department, Vissum Corporation Alicante, Alicante, Spain; 3 Division of Ophthalmology, Universidad Miguel Hernández, Alicante, Spain; 4 Keratoconus Unit of Vissum Corporation Alicante, Alicante, Spain; 5 Department of Refractive Surgery, Vissum Corporation Alicante, Alicante, Spain

**Keywords:** Keratoconus, *CAD*, Scheimpflug, Surface reconstruction, *Virtual model*

## Abstract

**Background:**

In case of significant imperfections on the cornea, data acquisition is difficult and a significant level of missing data could require the interpolation of important areas of the cornea, resulting in a very ambiguous model. The development of methods to define in vivo customised geometric properties of the cornea based only on real raw data is extremely useful to diagnose and assess the progression of diseases directly related to the corneal architecture. The present work tries to improve the prognostic of corneal ectasia creating a 3D customised model of the cornea and analysing different geometric variables from this model to determine which variables or combination of them could be defined as an indicator of susceptibility to develop keratoconus.

**Methods:**

A corneal geometric reconstruction was performed using zonal functions and retrospective Scheimpflug tomography data from 187 eyes of 187 patients. Morphology of healthy and keratoconic corneas was characterized by means of geometric variables. The performance of these variables as predictors of a new geometric marker was assessed and their correlations were analysed.

**Results:**

The more representative variable to classify the corneal anomalies related to keratoconus was posterior apex deviation (area under receiver operating characteristic curve > 0.899; p < 0.0001). However, the strongest correlations in both healthy and pathological corneas were provided by the metrics directly related to the thickness, as deviations of the anterior/posterior minimum thickness points.

**Conclusions:**

The presented morphogeometric approach based on the analysis and custom geometric modelling of the cornea demonstrates to be useful for the characterization and diagnosis of keratoconus disease, stating that geometrical deformation is an effective marker of the ectatic disease’s progression.

## Background

Keratoconus is an ectatic corneal disorder characterized by impaired vision and a poor quality of life. It is one of the leading indications for corneal transplantation. This pathology is characterised by a progressive corneal thinning and a structural weakening, resulting in corneal protrusion, irregular astigmatism and a gradual deterioration of the visual performance related to morphology changes in the corneal architecture. Several diagnostic criteria have been defined using a huge variety of techniques and technologies such as the classical keratoconus biomicroscopic signs, the conical protrusion and the infero-superior asymmetry [1].

As it has been demonstrated that small variations of the corneal morphology could induce important changes in the quality of life of the patients [2], recently other parameters such as geometric parameters of the keratoconic corneal surfaces have been analysed [3].

The geometric reconstruction of the corneal surface has experienced significant progress in recent years with the development of new technologies. In clinical practice the ophthalmologists use corneal topographers based on the Scheimpflug technology [4]. This diagnostic equipment gives a matrix of discrete points for the anterior and posterior surface of the cornea [5]. These raw data are not interpolated and are used to generate a geometric model with the modal methods called Zernike polynomials. These Zernike polynomials are defined for all discrete points of the anterior and posterior corneal surfaces for their reconstruction. However, in cases of pathological corneas, as for instance very aberrated corneas, it has been demonstrated that it is very difficult to define the Zernike polynomial order required to get the most relevant information about the corneal surfaces [5–7].

An alternative to these geometric models are the zonal methods called B-Spline functions [8]. These B-Spline functions divide the general raw data area used for the surface reconstruction into small more elemental subareas to get more flexible and more precise adjustment of the geometric model. These methods are widely used in Computer Aided Geometric Design (CAGD) for the virtual reconstruction of complex geometrical surfaces and its posterior analysis in different industrial fields [9, 10]. In Bioengineering, the zonal methods are used to generate virtual 3D models of the biological structures for different applications [11–16]. However, this technology is not extended to the Ophthalmology field, and it has only been used to generate customised models of the corneal biomechanics [3, 17–20]. In these cases, the raw data obtained were incomplete in the periphery and the limbus areas due to extrinsic errors during the data acquisition process, so the authors had to interpolate data in order to obtain a complete geometric model.

In the case of significant imperfections on the cornea, the data acquisition is difficult and a significant level of missing data could require the interpolation of important areas of the cornea resulting in a very ambiguous model. The development of methods to define in vivo customised geometric properties of the cornea based only on real raw data and without any kind of interpolation is extremely useful in clinical practice for the modelisation of pathological corneas [5].

**Fig. 1:**
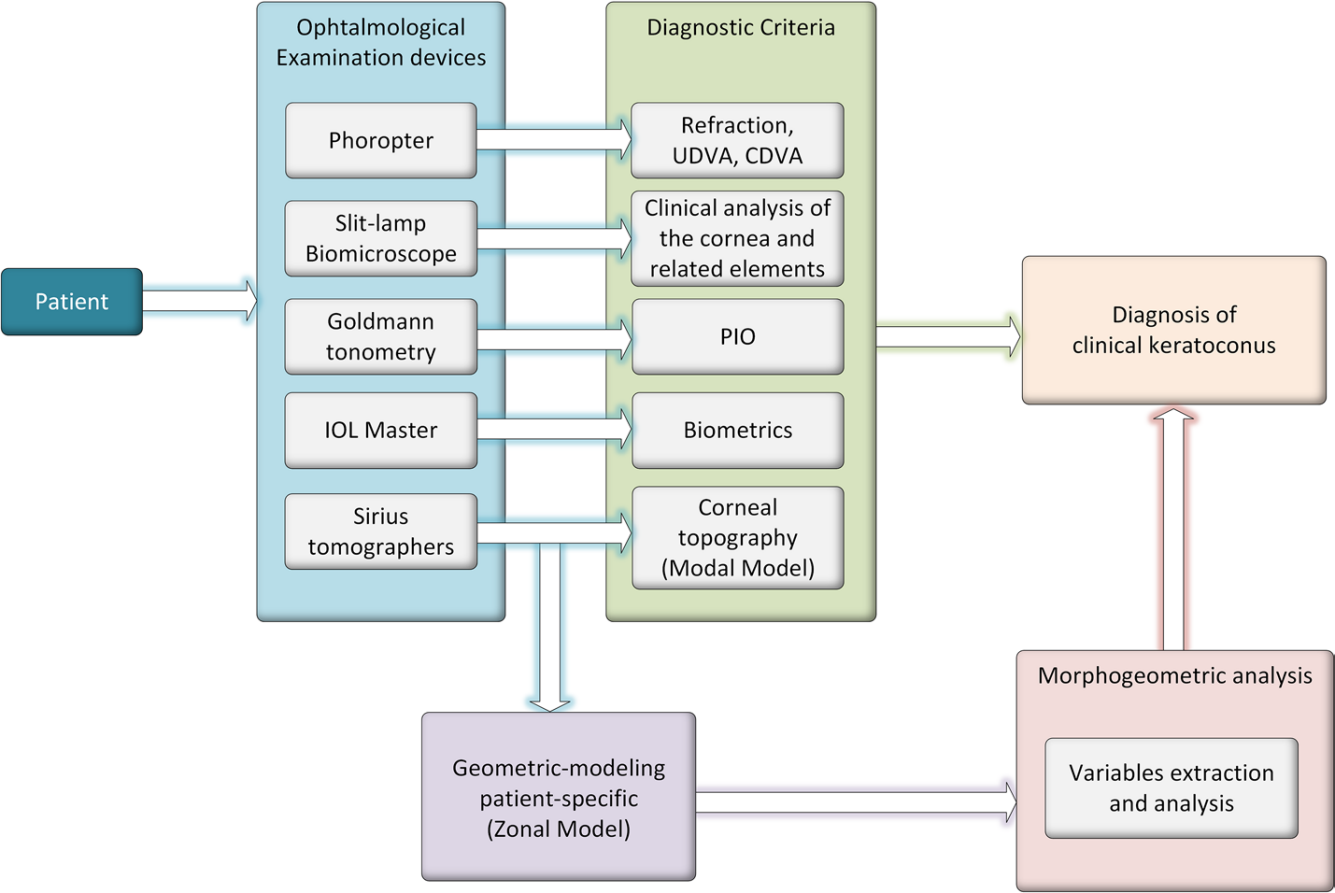
Use of patient-specific 3D modelling for the diagnosis of keratoconus

The present work tries to improve the prognostic of corneal ectasia using a new concept of diagnosis based on a geometric modelisation of the cornea. The Graphical Bioengineering technique used in this study will create a 3D customised model of the cornea and analyse different geometric variables of this model to determine, in a large-scale computational trial, which geometric variables or combination of variables could be defined as an indicator of susceptibility to develop keratoconus (Fig. 1).

A complete geometric analysis of the cornea of a huge number of patients has been carried out on raw data using Computer Aided Geometric Design with zonal methods and without any kind of interpolation. Different geometric variables have been defined for the 3D model of the cornea of each patient. These geometric variables have been analysed and compared according to the evolution of the pathology to define the most adequate one for the detection and prognostic of the evolution of the pathology.

The definition and analysis of the geometric profile of each cornea derived from the corneal pathology will enable to define the first changes related to the development of keratoconus and the evolution of the geometric profile according to the keratoconus severity, the corresponding loss of visual quality and the therapeutic treatments indicated.

## Methods

This was a retrospective study including 187 eyes of 187 patients ranging in age between 7 and 73 years old. Only one eye from each patient was randomly selected to avoid the interference in the analysis of the correlation that often exists between the two eyes of the same person. This study was conducted at Vissum Corporation in Alicante (Spain). Two groups of eyes were differentiated depending if the keratoconus disease was present or not: control group, including 124 healthy eyes, and keratoconus group, including 63 eyes with the diagnosis of keratoconus. The inclusion criterion for the control group was healthy eyes that did not meet the exclusion criteria and diagnosis according to the standard criteria for keratoconus diagnosis in the keratoconus group [21, 22], which is the presence of an asymmetric bowtie pattern in corneal topography, a value of 100 or higher of the KISA index, a central keratometry (K-value) with different cut-off values to keratoconus suspect (> 47.2 D), an inferior-superior asymmetry (I-S value) with a cut-off value of 1.4 D (difference between average inferior and superior corneal powers at 3 mm from the centre of the cornea), as well as other topographic indexes (SRAX, KSS, KPI, CLMI) and at least one keratoconus sign on slit-lamp examination, such as stromal thining, conical protusion on the cornea at the apex, Fleischer ring, Vogt striae or anterior stromal scar. Exclusion criteria in both groups were previous ocular surgery and any other active ocular disease. This study was approved by the Vissum Corporation ethics committee and was performed in accordance with the ethical standards laid down in the Declaration of Helsinki (Seventh revision, October 2013, Fortaleza, Brasil).

### Examination protocol

All patients underwent a complete eye examination including the following tests (Fig. 1): measurement of uncorrected (UDVA) and corrected distance visual acuity (CDVA), manifest refraction, slit-lamp biomicroscopy, PIO, biometrics (axial length, spherical equivalent, minimal corneal thickness, central corneal thickness, anterior chamber depth, white-to-white corneal diameter) and corneal topographic analysis. During this protocol, the Sirius system^®^ (CSO, Florence, Italy) was used, which is a noninvasive system for measuring and characterizing the anterior segment using a rotating Scheimpflug camera that generates images in three dimensions, with a dot matrix fine-meshed in the center due to the rotation. The images taken during the examination are digitalized in the main unit and transferred to a computer to be analyzed in detail. Gathered Sirius corneal topographies (data from other topographers such as Pentacam can also be handled [23]) are represented as discrete and finite set of spatial points (point cloud surfaces) in the form of two 31 × 256 matrices. Both matrices contain the polar coordinates representative of the anterior and posterior corneal surfaces. These data, used only in the first stage of the topographic data acquisition procedure, are called raw data [5, 23]. This warrantied that data were not interpolated or manipulated [23, 24], avoiding any proprietary reconstruction software from topographer’s manufacturer. All corneal topography files were exported in CSV format. Likewise, all cases were classified according to the Amsler-Krumeich grading system. All measurements were performed by the same experienced optometrists, performing three consecutive measurements and taking average values for posterior analysis.

### Diagnosis procedure

The procedure based on zonal methods and proposed in this article consists of two main stages: i) a geometrical modelling stage where the raw data provided by the corneal topographer is used to reconstruct a 3D geometrical model of the cornea using computational geometry techniques, and ii) a geometrical analysis stage where determined geometric variables are extracted from the model and analyzed to characterize the cornea.

#### First stage: geometrical modelling

**Fig. 2:**
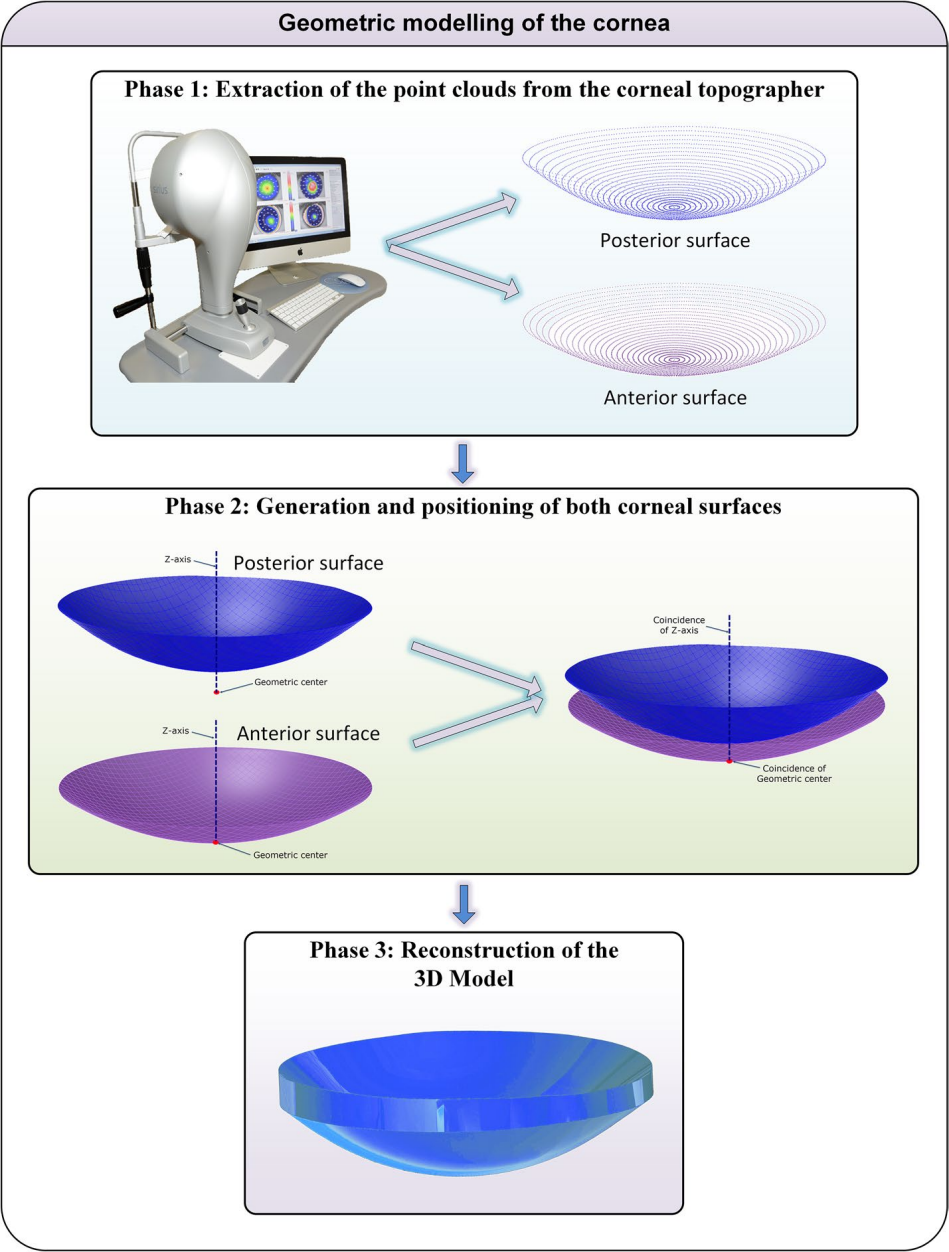
Geometric modelling process by using CAGD tools

The geometric reconstruction of the cornea was performed by executing the following steps (Fig. 2):

1. Extraction of the point clouds from the corneal topographer. The Sirius device used for this study provides two 3D point clouds that conform both the anterior and posterior corneal surfaces, respectively. However, due to Sirius topographer only provides spatial points data in Cartesian format for the anterior corneal surface (not for the posterior surface), data in polar format had to be exported to obtain data from both corneal surfaces. This data was given as a CSV (Comma Separated Values) table where every row represented a circle in the corneal map and every column represented a semi-meridian, giving 256 points for each radius. This way, each i-th row sampled a circle of i*0.2 mm radius on the map, and each j-th column sampled a semi-meridian in the direction of j*360/256° on the map, so each Z value of the matrix [i, j] represented the point P (i*0.2, j*360/256°) in polar coordinates. In order to perform these calculations, exported data were further formatted in Cartesian coordinates by the aid of an algorithm programmed in Matlab^®^.
2. Generation and positioning of both corneal surfaces. The two point clouds representing the corneal geometry were imported into the surface reconstruction software Rhinoceros^®^ v5.0, which uses a mathematical model to generate surfaces based on non-uniform rational B-spline (NURBS) with high accuracy. The surfaces that best fit the point clouds were generated with the Rhinoceros’ patch surface function, a reconstruction software option that fits a surface through given curves, meshes, point objects, and point clouds. For this research, this function tried to minimize the nominal distance between the 3D point clouds and the solution surfaces. For this objective, the function was configured by setting the sample point spacing at 256 (number of points for each data ring), the surface span planes at 255 for both u and v directions (the maximum number of span planes that the software permitted), and the stiffness of the solution surface at 10^−3^ (mm). This last parameter provides information on how much the best fit plane can be deformed in order to match the input points. This deviation can be calculated later by the software, providing a mean value of the distance error for the solution surface. During this study, an average distance error between the solution surface and the 3D point cloud of about 3.60 × 10^−4^ ± 6.43 × 10^−4^ mm was obtained. Using this procedure, anterior and posterior corneal surfaces were generated and engaged by their geometrical center and Z axis.
3. Solid modeling. After generation and positioning of both corneal surfaces, a third peripheral surface (the bonding surface between both sides in the Z-axis direction) was generated and then joined together to form a single surface. The surface reconstructed was then exported to the solid modeling software SolidWorks V 2013 (Dassault Systèmes, Vélizy-Villacoublay, France) to generate a solid model that is representative of the custom and actual geometry of each cornea.

A full process for the geometric reconstruction of a patient-specific cornea that comprises the three mentioned stages takes around 2–3 min.

#### Second stage: geometrical analysis

**Fig. 3:**
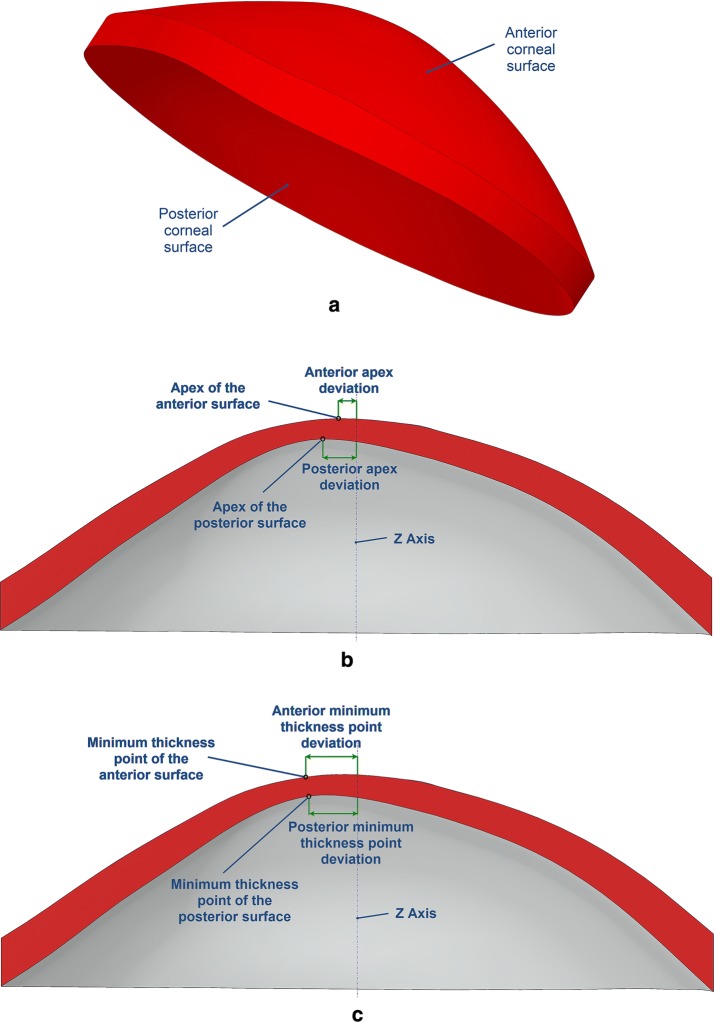
Geometric variables analyzed during the study: **a** Volume and area variables. **b** Anterior and posterior apex deviations. **c** Anterior and posterior minimum thickness point deviations

The resulting 3D solid model of the cornea was then used to perform an analysis of determined geometric variables (Fig. 3) that are representative of the corneal morphology (Table 1). These variables were later statistically analyzed in order to characterize the cornea.

**Table 1:** Geometric variables analyzed in the study

Geometric variable	Description
Total corneal volume (mm^3^)	Volume limited by front, back and peripheral surfaces of the solid model generated
Anterior corneal surface area (mm^2^)	Area of the front/exterior surface
Posterior corneal surface area (mm^2^)	Area of the rear/interior surface
Total corneal surface area (mm^2^)	Sum of anterior, posterior and perimetral corneal surface areas of the solid model generated
Anterior apex deviation (mm)	Average distance from the Z axis to the highest point (apex) of the anterior corneal surface
Posterior apex deviation (mm)	Average distance from the Z axis to the highest point (apex) of the posterior corneal surface
Anterior minimum thickness point deviation (mm)	Average distance in the XY plane from the Z axis to the minimum thickness point of the anterior corneal surface
Posterior minimum thickness point deviation (mm)	Average distance in the XY plane from the Z axis to the minimum thickness point of the posterior corneal surface

### Statistical analysis

A Kolmogorov–Smirnov test was run to assess the data engagement scores. According to this test and thereafter, a Student’s *t* test or U-Mann–Whitney Wilcoxon test was performed (depending on normality), in order to describe differences between normal and keratoconus groups in all the measurements proposed. Additionally, Kruskal–Wallis (K-W) and Effect Size (ES) tests were used to compare differences and to quantify the degree of change between groups according to Amsler-Krumeich Grading System (AK). For all statistical tests, the same level of significance was used (*p* < 0.05). Correlation coefficients (Pearson or Spearman depending if normality condition could be assumed) were used to assess the correlation between all different parameters. A linear regression was performed to quantify the strength of the correlation (R^2^) for both groups studied. A ROC curve analysis was performed in order to obtain the accuracy of the measurements. This ROC curve is a graphical plot that illustrates the performance of a binary classifier system as its discrimination threshold is varied. The curve is created by plotting the true positive rate against the false positive rate at various threshold settings. The accuracy of the test depends on how well the test separates the group being tested into those with and without the disease in question. The area under the ROC curve measures the accuracy: an area of 1 represents a perfect test, and an area of 0.5 represents a worthless test. A rough guide for classifying the accuracy of a diagnostic test is the traditional academic point system: excellent if 0.90–1; good if 0.80–0.90, fair if 0.70–0.80, and poor if 0.60–0.70. Statistical analyses were performed using Graphpad Prism version 6 sotfware (GraphPad Software, http://www.graphpad.com) and SPSS version 17.0 software (IBM, https://www-01.ibm.com).

## Results

From a total of 187 patients, this study included 124 healthy eyes that did not present any ocular pathology [25] and constituted by 69 females (55.6%) and 55 males (44.4%) ranged from 7 to 73 years old, and 63 eyes diagnosed with keratoconus in several grades [26] (53.9% in stage I, 31.7% in stage II, and 14.4% in the most extreme stages, III and IV) and formed by 34 females (53.9%) and 29 males (46.1%) ranged from 14 to 69 years old.

Table 2 shows the visual, refractive and morphological outcomes in the two groups of eyes analyzed during the study. No statistically significant differences between groups were found in axial length (*p *= 0.33, Student’s t-test), anterior chamber depth (*p *= 0.29, Mann–Whitney test), white to white corneal diameter (*p *= 0.71, Mann–Whitney test) and age (*p *= 0.09, Mann–Whitney test). Significant differences between groups were found in the remaining anatomical, refractive and visual parameters evaluated (*p *< 0.01 Student s t-test and Mann–Whitney test).

**Table 2:** Main clinical features of the two groups of eyes analyzed

Clinical features	Healthy N = 124	Keratoconic N = 63	*p* value (statistical test)
	Mean ± SD (range)	Mean ± SD (range)	
Age (years)	39.8 ± 18.6 (7 to 73)	37.02 ± 11.8 (14 to 69)	0.09 (Mann–Whitney)
Axial length (mm)	23.99 ± 1.60 (20.20 to 28.99)	24.20 ± 1.31 (21.82 to 26.50)	0.33 (Student’s t-test)
Corrected distance visual acuity	0.99 ± 0.09 (0.4 to 1.22)	0.80 ± 0.30 (0.12 to 1.09)	0.0001 (Mann–Whitney)
Sphere (D)	− 0.51 ± 3.29 (− 12.38 to 9.00)	− 2.01 ± 3.70 (− 17.00 to 3.00)	0.0001 (Mann–Whitney)
Cylinder (D)	− 0.80 ± 1 (− 5.75 to 0.00)	− 3.10 ± 2.40 (− 10.00 to 0.00)	0.0001 (Mann–Whitney)
Spherical equivalent (D)	− 0.99 ± 3.28 (− 12.50 to 8.20)	− 3.61 ± 3.89 (− 18.00 to 1.50)	0.0001 (Mann–Whitney)
Minimal corneal thickness (µm)	539.98 ± 32.70 (459.01 to 629.19)	439.78 ± 56.78 (315.08 to 549.08)	0.0001 (Mann–Whitney)
Central corneal thickness (µm)	543.07 ± 33.01 (464.10 to 633.84)	455.00 ± 49.12 (319.01 to 578.1)	0.0001 (Mann–Whitney)
Anterior chamber depth (mm)	2.89 ± 0.44 (1.79 to 3.70)	3.41 ± 0.4 (2.60 to 4.3)	0.29 (Mann–Whitney)
White to white corneal diameter (mm)	11.99 ± 0.48 (11.22 to 13.33)	12.06 ± 0.50 (11.09 to 13.71)	0.71 (Mann–Whitney)

On the other hand, all of the morphogeometric variables showed differences between normal and keratoconic eyes, as seen in Table 3. Total corneal volume presents higher values in healthy eyes (*p *< 0.0001), while anterior and posterior corneal surface areas are lower in the same subjects (*p *< 0.0001). This pattern of difference can be seen for most of the variables studied: healthy corneas have anterior and posterior apex deviations lower than keratoconic corneas (*p *< 0.0001), as occurs with the anterior and posterior minimum thickness point deviations.

**Table 3:** Morphogeometric variables measured in healthy and keratoconic corneas

Morphogeometric variables	Healthy N = 124	Keratoconic N = 63	p value (statistical test)
	Mean ± SD (range)	Mean ± SD (range)	
Total corneal volume (mm^3^)	25.90 ± 0.31 (25.59 to 26.21)	23.51 ± 0.48 (23.03 to 23.99)	0.0001 (Mann–Whitney)
Anterior corneal surface area (mm^2^)	43.13 ± 0.06 (43.07 to 43.19)	43.42 ± 0.13 (43.29 to 43.55)	0.0001 (Mann–Whitney)
Posterior corneal surface area (mm^2^)	44.31 ± 0.09 (44.22 to 44.40)	44.81 ± 0.21 (44.6 to 45.02)	0.0001 (Mann–Whitney)
Total corneal surface area (mm^2^)	104.02 ± 0.29 (103.73 to 104.31)	103.68 ± 0.43 (103.25 to 104.11)	0.0001 (Mann–Whitney)
Anterior apex deviation (mm)	0.0003 ± 0.0002 (0.0001 to 0.0005)	0.0090 ± 0.0035 (0.0055 to 0.0125)	0.0001 (Mann–Whitney)
Posterior apex deviation (mm)	0.0771 ± 0.0128 (0.0643 to 0.0899)	0.1902 ± 0.029 (0.1603 to 0.2201)	0.0001 (Mann–Whitney)
Anterior minimum thickness point deviation (mm)	0.875 ± 0.052 (0.823 to 0.927)	1.101 ± 0.19 (0.912 to 1.291)	0.0001 (Mann–Whitney)
Posterior minimum thickness point deviation (mm)	0.811 ± 0.051 (0.772 to 0.862)	0.960 ± 0.20 (0.76 to 1.16)	0.0001 (Mann–Whitney)

Outcomes according to keratoconus severity are shown in Table 4 (clinical features) and Table 5 (morphogeometric parameters), where comparisons are established according to the AK grading system. Additionally, note that calculated effect sizes for each disease stage allows quantifying the degree of change, which is higher for stages III and IV in all of the variables, becoming more evident with the progress of the disease.

**Table 4:** Main clinical features measured in healthy and keratoconic corneas

Clinical features	Normal	Stage I	Stage II	Stage III–IV	p (KW test)
	Mean ± SD (range)	Mean ± SD (range)	Mean ± SD (range)	Mean ± SD (range)	
Age (years)	39.8 ± 18.6 (7 to 73)	36.02 ± 10.2 (17 to 69)	35.1 ± 16.88 (14 to 62)	27.12 ± 5.01 (19 to 40)	0.31
(ES)	–	1.30	1.41	3.62	
Axial length (mm)	23.99 ± 1.60 (20.20 to 28.99)	24.08 ± 1.40 (21.80 to 26.44)	24.20 ± 1.36 (22.30 to 27.4)	24.69 ± 1.42 (23.69 to 26.66)	0.79
(ES)	–	− 1.20	− 1.69	− 3.79	
Corrected distance visual acuity	0.99 ± 0.09 (0.4 to 1.22)	0.03 ± 0.66 (-0.10 to 0.19)	0.20 ± 0.18 (0.01 to 0.51)	0.49 ± 0.24 (0.16 to 0.74)	0.0001
(ES)	–	− 0.09	− 1.51	− 2.21	
Sphere (D)	− 0.51 ± 3.29 (− 12.38 to 9.00)	− 0.88 ± 1.38 (− 4.61 to 1.14)	− 2.30 ± 3.44 (− 10.99 to 3.11)	− 3.8 ± 6.70 (− 15.00 to 0.01)	0.0001
(ES)	–	0.29	0.41	0.12	
Cylinder (D)	− 0.80 ± 1 (− 5.75 to 0.00)	− 2.10 ± 1.33 (− 4.75 to 0.5)	− 3.71 ± 2.88 (− 10.5 to 0.5)	− 4.4 ± 3.88 (− 8.0 to − 2.5)	0.0001
(ES)	–	− 1.12	− 1.48	− 4.22	
Spherical equivalent (D)	− 0.99 ± 3.28 (− 12.50 to 8.20)	− 1.89 ± 1.49 (− 8 to 0.74.50)	− 4.90 ± 3.29 (− 12.05 to 1.20)	− 6.75 ± 5.40 (− 18.5 to 0.50)	0.0001
(ES)	–	− 0.88	− 1.68	− 3.44	
Minimal corneal thickness (µm)	539.98 ± 32.70 (459.01 to 629.19)	469.54 ± 33.48 (390.12 to 528.30)	441.08 ± 41.22 (369.54 to 535.11)	354.12 ± 43.12 (338.41 to 492.12)	0.0001
(ES)	–	− 0.10	− 1.28	− 4.01	
Central corneal thickness (µm)	543.07 ± 33.01 (464.10 to 633.84)	474.07 ± 38.01 (392.01 to 578.1)	451.2 ± 40.11 (371.2 to 540.12)	361.24 ± 51.2 (319.01 to 532.1)	0.0001
(ES)	–	− 0.18	− 1.41	− 3.89	
Anterior chamber depth (mm)	2.89 ± 0.44 (1.79 to 3.70)	3.30 ± 0.31 (2.68 to 4.01)	3.40 ± 0.5 (2.71 to 4.22)	3.82 ± 0.31 (3.50 to 4.23)	0.0001
(ES)	–	0.10	0.12	0.22	
White to white corneal diameter (mm)	11.99 ± 0.48 (11.22 to 13.33)	12.00 ± 0.48 (11.19 to 13.38)	12.04 ± 0.47 (11.09 to 13.44)	12.07 ± 0.51 (11.11 to 13.71)	0.0001
(ES)	–	0.05	0.09	0.1	

*ES* effect size

**Table 5:** Morphogeometric variables measured in healthy and keratoconic corneas

Morphogeometric variables	Normal	Stage I	Stage II	Stage III–IV	p (KW test)
	Mean ± SD (range)	Mean ± SD (range)	Mean ± SD (range)	Mean ± SD (range)	
Total corneal volume (mm^3^)	25.90 ± 0.31 (25.59 to 26.21)	23.51 ± 0.48 (23.03 to 23.99)	23.09 ± 0.59 (22.5 to 23.68)	20.01 ± 2.88 (17.13 to 22.89)	0.0001
(ES)	–	1.21	1.39	3.41	
Anterior corneal surface area (mm^2^)	43.13 ± 0.06 (43.07 to 43.19)	43.42 ± 0.13 (43.29 to 43.55)	43.50 ± 0.17 (43.33 to 43.67)	44.31 ± 0.21 (44.1 to 44.52)	0.0001
(ES)	–	− 1.16	− 1.66	− 4.79	
Posterior corneal surface area (mm^2^)	44.31 ± 0.09 (44.22 to 44.40)	44.81 ± 0.21 (44.6 to 45.02)	44.99 ± 0.22 (44.7 to 45.21)	45.39 ± 0.32 (45.07 to 45.71)	0.0001
(ES)	–	− 0.101	− 1.49	− 4.21	
Total corneal surface area (mm^2^)	104.02 ± 0.29 (103.73 to 104.31)	103.68 ± 0.43 (103.25 to 104.11)	103.59 ± 0.39 (103.2 to 103.98)	103.53 ± 0.52 (103.01 to 104.05)	0.0001
(ES)	–	0.31	0.20	0.29	
Anterior apex deviation (mm)	0.0001 ± 0.00001 (0.0000 to 0.0002)	0.006 ± 0.0021 (0.0039 to 0.0081)	0.009 ± 0.0035 (0.0055 to 0.0125)	0.012 ± 0.004 (0.008 to 0.016)	0.0001
(ES)	–	− 1.11	− 1.39	− 5.70	
Posterior apex deviation (mm)	0.0771 ± 0.0128 (0.0643 to 0.0899)	0.17 ± 0.029 (0.141 to 0.199)	0.201 ± 0.03 (0.171 to 0.231)	0.237 ± 0.051 (0.186 to 0.288)	0.0001
(ES)	–	− 1.19	− 1.40	− 1.23	
Anterior minimum thickness point deviation (mm)	0.901 ± 0.58 (0.429 to 2.201)	0.99 ± 0.21 (0.052 to 0.210)	1.066 ± 0.25 (0.533 to 3.22)	0.299 ± 0.3 (0.244 to 0.568)	0.0001
(ES)	–	− 0.69	− 0.77	1.88	
Posterior minimum thickness point deviation (mm)	0.780 ± 0.013 (0.38 to 2.11)	0.961 ± 0.033 (0.541 to 1.89)	0.989 ± 0.04 (0.450 to 2.899)	0.360 ± 0.047 (0.188 to 0.529)	0.0001
(ES)	–	− 0.7	− 0.73	1.70	

*ES* effect size

Table 6 summarizes the statistically significant correlations between all the modeled morphogeometric variables for the normal group, and Table 7 shows the significant correlations for the KC group.

**Table 6:** Correlations between modeled morphogeometric variables for the healthy group

Variables	Total corneal volume	Anterior corneal surface area	Posterior corneal surface area	Total corneal surface area	Anterior apex deviation	Posterior apex deviation	Anterior minimum thickness point deviation	Posterior minimum thickness point deviation
Total corneal volume	r = 1	r = 0.010 (p = 0.911)	r = − 0.271 (p = 0.008)	r = ***0.953*** (p = 0.000)	r = − 0.041 (p = 0.690)	r = 0.211 (p = 0.39)	r = − 0.45 (p = 0.664)	r = − 0.029 (p = 0.758)
Anterior corneal surface area	r = 0.01 (p = 0.911)	r = 1	r = − 0.801 (p = 0.000)	r = 0.374 (p = 0.000)	r = − 0.062 (p = 0.550)	r = 0.019 (p = 0.855)	r = 0.072 (p = 0.486)	r = 0.046 (p = 0.655)
Posterior corneal surface area	r = − 0.271 (p = 0.008)	r = − 0.801 (p = 0.000)	r = 1	r = 0.702 (p = 0.000)	r = − 0.092 (p = 0.370)	r = 0.127 (p = 0.217)	r = 0.025 (p = 0.810)	r = − 0.000 (p = 0.999)
Total corneal surface area	r = ***0.953*** (p = 0.000)	r = 0.374 (p = 0.000)	r = 0.702 (p = 0.000)	r = 1	r = − 0.075 (p = 0.473)	r = 0.226 (p = 0.27)	r = − 0.29 (p = 0.783)	r = − 0.030 (p = 0.761)
Anterior apex deviation	r = − 0.041 (p = 0.690)	r = − 0.062 (p = 0.550)	r = − 0.092 (p = 0.370)	r = − 0.075 (p = 0.473)	r = 1	r = 0.015 (p = 0.88)	r = 0.089 (p = 0.389)	r = 0.082 (p = 0.427)
Posterior apex deviation	r = 0.211 (p = 0.39)	r = 0.019 (p = 0.855)	r = 0.127 (p = 0.217)	r = 0.226 (p = 0.27)	r = 0.015 (p = 0.88)	r = 1	r = 0.150 (p = 0.141)	r = 0.169 (p = 0.101)
Anterior minimum thickness point deviation	r = − 0.45 (p = 0.664)	r = 0.072 (p = 0.486)	r = 0.025 (p = 0.810)	r = − 0.29 (p = 0.783)	r = 0.089 (p = 0.389)	r = 0.150 (p = 0.141)	r = 1	r = ***0.996*** (p = 0.000)
Posterior minimum thickness point deviation	r = − 0.029 (p = 0.758)	r = 0.046 (p = 0.655)	r = − 0.000 (p = 0.999)	r = − 0.030 (p = 0.761)	r = 0.082 (p = 0.427)	r = 0.169 (p = 0.101)	r = ***0.996*** (p = 0.000)	r = 1

BoldItalic indicates strong correlation of the variables

**Table 7:** Correlations between modeled morphogeometric variables for the KC group

Variables	Total corneal volume	Anterior corneal surface area	Posterior corneal surface area	Total corneal surface area	Anterior apex deviation	Posterior apex deviation	Anterior minimum thickness point deviation	Posterior minimum thickness point deviation
Total corneal volume	r = 1	r = − 0.381 (p = 0.004)	r = − 0.182 (p = 0.185)	r = 0.610 (p = 0.000)	r = − 0.284 (p = 0.34)	r = − 0.198 (p = 0.887)	r = − 0.24 (p = 0.108)	r = − 0.226 (p = 0.098)
Anterior corneal surface area	r = − 0.381 (p = 0.004)	r = 1	r = ***0.921*** (p = 0.000)	r = 0.421 (p = 0.000)	r = 0.544 (p = 0.000)	r = 0.387 (p = 0.003)	r = − 0.073 (p = 0.601)	r = − 0.069 (p = 0.620)
Posterior corneal surface area	r = − 0.182 (p = 0.185)	r = ***0.921*** (p = 0.000)	r = 1	r = 0.668 (p = 0.000)	r = 0.479 (p = 0.000)	r = 0.349 (p = 0.008)	r = − 0.170 (p = 0.225)	r = − 0.159 (p = 0.236)
Total corneal surface area	r = 0.610 (p = 0.000)	r = 0.421 (p = 0.000)	r = 0.668 (p = 0.000)	r = 1	r = 0.133 (p = 0.329)	r = 0.219 (p = 0.105)	r = − 0.325 (p = 0.015)	r = − 0.328 (p = 0.18)
Anterior apex deviation	r = − 0.284 (p = 0.34)	r = 0.544 (p = 0.000)	r = 0.479 (p = 0.000)	r = 0.133 (p = 0.329)	r = 1	r = 0.540 (p = 0.000)	r = 0.006 (p = 0.974)	r = 0.21 (p = 0.921)
Posterior apex deviation	r = − 0.198 (p = 0.887)	r = 0.387 (p = 0.003)	r = 0.349 (p = 0.008)	r = 0.349 (p = 0.008)	r = 0.219 (p = 0.105)	r = 1	r = 0.321 (p = 0.020)	r = 0.319 (p = 0.018)
Anterior minimum thickness point deviation	r = − 0.24 (p = 0.108)	r = − 0.073 (p = 0.601)	r = − 0.170 (p = 0.225)	r = − 0.325 (p = 0.015)	r = 0.006 (p = 0.974)	r = 0.321 (p = 0.020)	r = 1	r =*** 0.999*** (p = 0.000)
Posterior minimum thickness point deviation	r = − 0.226 (p = 0.098)	r = − 0.069 (p = 0.620)	r = − 0.159 (p = 0.236)	r = − 0.328 (p = 0.18)	r = 0.21 (p = 0.921)	r = 0.319 (p = 0.018)	r =*** 0.999 ***(p = 0.000)	r = 1

BoldItalic indicates strong correlation of the variables

In the healthy eyes group two strong correlations (above 0.9) have been achieved: on one hand, between total corneal volume and total corneal surface area (*r *= 0.953; *p *= 0.000), and on the other hand between anterior and posterior minimum thickness point deviations (*r *= 0.996; *p *= 0.000).

In the same way, two strong correlations (above 0.9) have also been found in the keratoconic eyes group for several analysed morphogeometric variables: between anterior and posterior corneal surface areas (*r *= 0.921; *p *= 0.000), and between anterior and posterior minimum thickness point deviations (*r *= 0.999; *p *= 0.000).

**Fig. 4:**
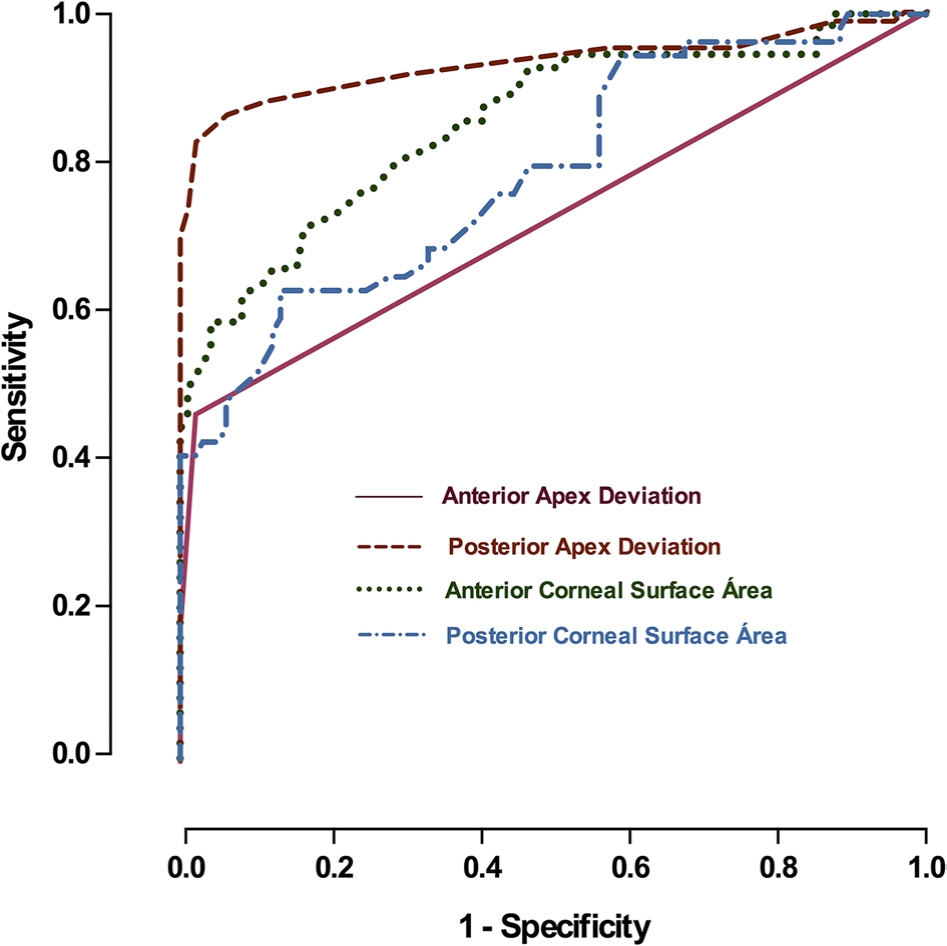
A ROC analysis modelling the sensitivity versus 1-specificity for variables predicting the existence of keratoconus disease using geometrical custom modelling of the cornea

The predictive value of the modeled variables was established by a ROC analysis (Fig. 4). From the several geometric variables analyzed during the study, the variables that achieved the best results in the diagnosis of the disease with an area under the ROC curve (AUROC) above 0.7 were the following four: *anterior corneal surface area* (Fig. 3a) (area: 0.853, *p* < 0.0001, std. error: 0.040, 95% CI 0.762–0.919), with a cutoff value of 43.07 mm^2^, and an associated sensitivity and specificity of 90.27% and 60.01%, respectively; *the posterior corneal surface area* (Fig. 3a) (area: 0.813, *p* < 0.0001, std. error: 0.039, 95% CI 0.719–0.891), with a cutoff value of 44.18 mm^2^, and an associated sensitivity and specificity of 91.08% and 44.17%, respectively; *anterior apex deviation* (Fig. 3b) (area: 0.742, *p* < 0.0001, std. error: 0.059, 95% CI 0.641–0.875), with a cutoff value of 0.0013 mm, and an associated sensitivity and specificity of 72.02% and 92.01%, respectively; *posterior apex deviation* (Fig. 3b) (area: 0.899, *p* < 0.0001, std. error: 0.041, 95% CI 0.800–0.964), with a cutoff value of 0.0855 mm, and an associated sensitivity and specificity of 91.28% and 73.07%, respectively. By contrast, there are two morphogeometric variables that present an area under the ROC curve below but very close to 0.7: anterior minimum thickness point deviation (area: 0.688, *p* < 0.0011, std. error: 0.05788, 95% CI 0.5759–0.7901) and posterior minimum thickness point deviation (area: 0.691, *p* < 0.0010, std. error: 0.05888, 95% CI 0.5760–0.7978.

Thus, according to the area under the curve variable calculated for the analysed parameters, it was concluded that the parameter that provides a higher rate of discrimination between normal corneas and corneas with keratoconus is *Posterior apex deviation*. Nevertheless, there are other relevant statistical differences between healthy and diseased eyes, and most of variables studied differ between groups, making it possible to differentiate with high sensitivity and specificity healthy corneas from those patients diagnosed with keratoconus.

## Discussion

The introduction of advanced imaging technologies in clinical practice, such as scanning-slit topography or Scheimpflug tomography has allowed the clinician to perform a more consistent and precise diagnosis of forms of keratoconus, helping to characterize the global geometry of the corneal surface [4]. Several parameters, such as pachymetry, corneal volume or corneal wavefront aberrations, are used in the batteries tests for corneal ectasia diagnosis, providing a characterization of the underlying morphogeometric alteration [21]. However, the geometrical characterization indices proposed by these devices are not easily compatible between different tomographers, generating confusion in the Ophthalmic Community [27–29].

On the other hand, up to date, the ability of a specific geometric model to capture diseases on human corneas has not been assessed, either the correlation values between morphogeometric parameters on both normal and pathological groups. Specifically, this computational study provides insight into the complex clinical problem of diagnosing corneal ectatic diseases.

It is well known that the mechanical response of any deformable system is affected by its geometry and material properties. When geometry is fully characterized, it is possible to set up a geometric model of the system, which may be used to analyze the geometric response under original conditions. In this case, conditions are defined by the rupture of the geometric balance due to the existence of a biomechanical weakening, as happens in the keratoconus disease. Keratoconus is a disorder characterized by a progressive thinning of the cornea, which is physically presented in its structure as a protrusion or cone type focal curving that entails a redistribution of its pachymetry and some changes in the anatomical morphology of its surfaces [21].

Other aspect to be taken into account is the geometric characterization based on raw data, which has been previously used by some authors in the Corneal Biomechanics field [20, 23] and for diagnosis of corneal diseases [30]. However, these studies resort to data interpolation to obtain a specific model for each patient. Geometric modeling based on raw data that has not been treated by any internal algorithm of the topographer enables an accurate clinical characterization of the human cornea basing on perfectly defined morphological variables in the field of graphic bioengineering.

During this study, two different types of parameters were analysed: (i) clinical features and (ii) morphogeometric variables. During the first analysis, no significant differences in age were found between the different grades of keratoconus (*p *= 0.31). However, statistically significant differences between the healthy group and the different grades of keratoconus were found for refractive and biometric results (*p *= 0.0001).

In the second analysis, significant differences were found for the morphogeometric variables between the health and disease groups, as well as between the different evolution grades of the disease. This fact reveals that, even though the curvature radii are smaller on both surfaces in the KC group, the morphogeometric variables register the tendency of the cornea to develop and to maintain its structure in form of meniscus until the most advanced grades of the disease, in which the relationship between both corneal surfaces is significantly modified with a greater increase of the curvature (distance between the corneal apex and the geometric centre of the cornea) of the posterior corneal surface compared to the anterior corneal surface. This trend is on line with the tendency reported by other studies [31], where the posterior-anterior radius of curvature is analysed for groups with healthy eyes and keratoconic eyes according to the Amsler-Krumeich classification. Specifically for keratoconus, in this study a significant increase of the curvature of the posterior corneal surface was observed with respect to the anterior surface (*p *< 0.01) in the most advanced disease stage. According to these findings, the severity grade of the keratoconus disease seems to be related to the geometry of the anterior and posterior corneal surfaces. This fact could be associated with the alteration of the biomechanical properties existing in a keratoconic cornea, and more concretely in the most advanced grades of the disease. Theoretically, this biomechanical weakness can make the cornea to be subject of deformation due to the intraocular pressure, affecting largely to the posterior surface curvature.

Regarding the ROC analysis, the posterior apex deviation showed the highest discriminant coefficient (AUROC, 0.899). In accordance with a previous publication [32, 33], the posterior curvature might influence on the visual function. We hypothesize that the magnitude of the posterior apex deviation represents the best performance to recognize the geometric profile of the KC stage where the visual acuity starts to impair. Furthermore, we also found a satisfactory level of discriminative ability for anterior corneal surface area (AUROC, 0.853), posterior corneal surface area (AUROC, 0.813) and the anterior apex deviation (AUC, 0.742).

This study also assessed the relationship of the geometrical pattern of both anterior and posterior corneal surfaces. Other authors, for a different purpose, found strong statistical correlations between the anterior and posterior shape factors for keratoconic corneas. Regarding the minimum thickness point deviations from both corneal surfaces, significant differences between groups were found. Interestingly, the strongest correlation value yielded in this investigation for keratoconic eyes was between the anterior and posterior minimun thickness point deviations (R^2^ = 0.999; *p* = 0.000).

## Conclusions

This study relies on the use of a reduced number of geometrical parameters obtained from modeling tests of the cornea: anterior corneal surface area, posterior corneal surface area, anterior apex deviation and posterior apex deviation. These variables are sufficient to prove that the variability of the geometric response of human corneas is definitely related to diagnosis of the disease. This method is simplified and more integrative than current diagnostic systems, which analyse separately the anterior and posterior surfaces of the cornea. This observation has a quite relevant implication in view of the prediction of the response to refractive surgery, i.e. the knowledge of the sole geometry is enough to feed keratoconus diagnosis.

The main suggestion derived from this study is to give high priority to the development of non-invasive testing methods that are able to provide through inverse analysis the patient-specific parameters of a sufficiently realistic geometric model of the corneal morphology, which can be obtained with the aid of Computer Aided Geometric Design tools.

This method will allow improving the detection and effects of therapeutic methods used for keratoconus and other corneal ectatic diseases such as post-lasik ectasia. Early studies, currently under publication, have demonstrated the effectiveness of this approach in the early detection of subclinical keratoconus. In a close future, thanks to the analyses of the objective data related to the geometric effect of the intracorneal rings implanted, customized nomograms for the implantation of Intra-Corneal Rings will be developed. Later, the analysis of the correlation between the geometric, visual, biomechanical and clinical effects of intracorneal implants in ectatic corneas will allow the development of new therapies and new concepts of corneal implants. The geometric modeling developed will also allow assessing more accurately the outcomes of the corneal crosslinking techniques and its effectiveness in slowing the development of keratoconus.
